# Role of *ACE* and *PAI-1* Polymorphisms in the Development and Progression of Diabetic Retinopathy

**DOI:** 10.1371/journal.pone.0144557

**Published:** 2015-12-14

**Authors:** Saba Saleem, Aisha Azam, Sundus Ijaz Maqsood, Irfan Muslim, Shaheena Bashir, Nosheen Fazal, Moeen Riaz, Syeda Hafiza Benish Ali, Muhammad Khizar Niazi, Mazhar Ishaq, Nadia Khalida Waheed, Raheel Qamar, Maleeha Azam

**Affiliations:** 1 Department of Biosciences, COMSATS Institute of Information Technology, Islamabad, Pakistan; 2 Institute of Ophthalmology, Mayo Hospital, Lahore, Pakistan; 3 Royal College of Ophthalmologists, Mersey Deanery, United Kingdom; 4 Armed Forces Institute of Ophthalmology, Rawalpindi, Pakistan; 5 Army Medical College, Rawalpindi, Pakistan; 6 Tufts University Medical School, Boston, Massachusetts, United States of America; 7 Al-Nafees Medical College and Hospital, Isra University, Islamabad, Pakistan; 8 Pakistan Academy of Sciences, Islamabad, Pakistan; Cedars-Sinai Medical Center; UCLA School of Medicine, UNITED STATES

## Abstract

In the present study we determined the association of angiotensin converting enzyme (*ACE*) and plasminogen activator inhibitor-1 (*PAI-1*) gene polymorphisms with diabetic retinopathy (DR) and its sub-clinical classes in Pakistani type 2 diabetic patients. A total of 353 diabetic subjects including 160 DR and 193 diabetic non retinopathy (DNR) as well as 198 healthy controls were genotyped by allele specific polymerase chain reaction (PCR) for *ACE* Insertion/Deletion (ID) polymorphism, rs4646994 in intron 16 and *PAI*-*1* 4G/5G (deletion/insertion) polymorphism, rs1799768 in promoter region of the gene. To statistically assess the genotype-phenotype association, multivariate logistic regression analysis was applied to the genotype data of DR, DNR and control individuals as well as the subtypes of DR. The *ACE* genotype ID was found to be significantly associated with DR (p = 0.009, odds ratio (OR) 1.870 [95% confidence interval (CI) = 1.04–3.36]) and its sub-clinical class non-proliferative DR (NPDR) (p = 0.006, OR 2.250 [95% CI = 1.098–4.620]), while *PAI* polymorphism did not show any association with DR in the current cohort. In conclusion in Pakistani population the *ACE* ID polymorphism was observed to be significantly associated with DR and NPDR, but not with the severe form of the disease i.e. proliferative DR (PDR).

## Introduction

Diabetic Retinopathy (DR) is one of the most damaging microvascular complication of diabetes mellitus and remains a major cause of visual morbidity among the developed and under developed countries [1, 2]. Being a progressive disease, it severely damages the cellular and the structural components of the retinal vasculature resulting in vision impairment, while often leading to blindness [2]. Nearly all patients with type 1 diabetes mellitus (T1DM) and more than 60% patients with type 2 diabetes mellitus (T2DM) develop retinopathy in the first two decades of disease onset [3]. In developing countries, DR is considered as the major cause of blindness in people of working age and is characterized by hyperglycemia, thickening of the basement membrane accompanying loss of pericytes, microaneurysms, dysfunction of the endothelial cell, microvascular infarcts and neovascularization, which can eventually lead to loss of vision due to hemorrhages and detachment of the retina [1]. Clinically DR manifests as a mild non-proliferative condition with augmented vascular permeability, which progresses to moderate and severe nonproliferative DR (NPDR) with vascular closure that then advances to proliferative DR (PDR), where new blood vessels are formed in the retina and posterior surface of the vitreous. Macular edema, retinal thickening from leaky blood vessels, can develop at all stages of retinopathy. These conditions ultimately lead to irreversible vision loss [1].

Angiotensin converting enzyme (ACE), which is a component of the renin-angiotensin system (RAS) plays an important role in the regulation of systemic and renal circulation by converting angiotensin I into vasoconstrictor molecule angiotensin II [4]. A number of studies have reported that patients suffering from proliferative retinopathy have high circulating levels of ACE, which implies that elevated serum ACE levels might be a possible risk factor in damaging retinal vascular apparatus in subjects suffering from diabetes [5]. Another factor plasminogen activator inhibitor-1 (PAI-1) has a potent antifibrinolytic activity in the plasmin regulated proteolytic cascade where it plays a vital role in inhibition of fibrinolysis and degradation of extracellular matrix [6]. A population-based study in Pima Indians suffering from T2DM has demonstrated that the 4G polymorphism in the promoter region of *PAI*-1 was associated with high risk of development of retinopathy, as elevated serum levels of PAI-1 result in dysfunctioning of the endothelium, which could lead to thickening of blood vessels [7].

Despite a number of studies the evidence of association of these genes in the development of different multifactorial diseases including diabetes-induced retinopathy remains inconsistent, not only because of the involvement of multifactorial pathways in the pathogenesis of diabetes but also due to genetic diversity among different populations worldwide. Therefore, the present case control association study was conducted to investigate the role of *ACE* and *PAI-1* polymorphisms in the onset of DR in Pakistani type 2 diabetics, which resulted in the identification of a significant association of a studied polymorphism with the disease.

## Methods

### Sample collection and DNA isolation

The cases were clinically diagnosed and sampled from Shifa International Hospital (Islamabad), Railway Hospital (Rawalpindi), Armed Forces Institute of Ophthalmology (Rawalpindi) and Mayo Hospital (Lahore). The current study was approved by the Department of Biosciences Ethics Review Board, COMSATS Institute of Information Technology, Islamabad, Pakistan. Before taking the blood samples, each participant was informed about the purpose of the study and a written consent in accordance with the guidelines of the Helsinki Declaration was taken. All the patients who participated in the present study belonged to the same ethnic group therefore there was no ethnicity bias in the data. The controls were sampled from the general population to which the cases belonged.

The current study was a case-control association analysis. All cases were initially diagnosed for T2DM by a qualified endocrinologist and had T2DM for more than 10 years. Patients were subjected to detailed eye examination (ophthalmoscopy and funduscopy) to assess retinopathy. The patients who were negative and positive for retinopathy were classified as DNR and DR, respectively. The inclusion criteria for the patients suffering from T2DM was according to the American Diabetes Association: age 18–75 years, fasting plasma glucose level ≥ 126mg/dl, random plasma glucose concentration ≥ 200mg/dl, serum creatinine concentration ≤ 2.0 mg/dl and glycated hemoglobin 6.5% or higher [8]. The selected patients were then subjected to detailed ocular examination including fundus examination with direct and indirect ophthalmoscope and also with 78D lens on the Slit lamp. Patients with DR were broadly distributed into two groups as PDR and NPDR on the basis of ETDRS (Early Treatment Diabetic Retinopathy) classification. According to ETDRS patients with NPDR having very mild form with only microaneurysms as well as with very severe form having severe retinal hemorrhages in all four quadrants, about 20 medium-large per quadrant, significant venous beading in two quadrants, and moderate IRMA in one or more quadrant were selected. Based on the diagnostic criteria a total of 90 individuals with a mean age of 54 years were characterized as NPDR. The PDR patients (n = 70, mean age = 56 years) with mild-moderate form of the disease having new vessels on disc (NVD) or new vessels elsewhere (NVE), and high-risk form having new vessels on disc (NVD) about one third disc area, or any NVD with vitreous or preretinal hemorrhage or NVE greater than half disc area with vitreous or preretinal hemorrhage were sampled. In addition patients having advanced diabetic retinopathy with tractional retinal detachment were also included in PDR group. Out of the sampled 353 T2DM cases, 160 [77 (48%) males and 83 (51.8%) females] were suffering from DR and 193 [94 (48.7%) males and 99 (51.3%) females] had DNR. While 198 individuals (100 (50.5%) males and 98 (49.5%) females), negative for any type of diabetes, hypertension, myocardial infarction, cancer and retinopathy independent of diabetes were included in the study as unaffected controls (Table 1).

**Table 1:** Demographic data of the patients genotyped for *ACE* and *PAI* polymorphisms in the present study.

Cases	Mean age (years)	Fasting glucose (gm/ dL)	HbA1c levels (% age)	Retinal changes
T2DM (DNR+DR)	48	>140	>7–8	—
DNR	45	>140	>7–8	—
DR (NPDR+PDR)	56	>140	>7–8	Microaneurysms, hard exudates, macular edema, neovascularization
NPDR	54	>140	>7–8	Microaneurysms, retinal ischemia, hemorrhages, hard exudates
PDR	56	>140	>7–8	Macular edema, boat shaped pre-retinal hemorrhage, neovascularization

T2DM, type 2 diabetes mellitus; DNR, diabetic non-retinopathy; DR, diabetic retinopathy; NPDR, non-proliferative diabetic retinopathy; PDR, proliferative diabetic retinopathy

Blood samples of patients and controls were collected in 8.5 ml BD vacutainer tubes (1 Becton Drive, Franklin Lakes, NJ) containing ethylene diamaine tetra-acetic acid (EDTA) as an anti coagulant. DNA extraction from the lymphocytes was carried out using standard organic phenol-chloroform method [9].

### Genotyping

The *ACE* Insertion/Deletion (ID) polymorphism rs4646994 in intron 16 and *PAI-1* 4G/5G (deletion/insertion) polymorphism rs1799768 in promoter region were genotyped as described previously by Anderson et al. [10] and Ahmed et al. [11], respectively.

### Statistical Analysis

The genotyped data for both *ACE* (ID) and *PAI-1* (4G/5G) polymorphism were analyzed statistically. The genotype and allele frequencies were compared among cases and controls by using z-test. To assess the association of disease with genotype, logistic regression analysis was performed, while adjusting for age, gender and sub-clinical classes including NPDR and PDR. The analyses were performed using R software (R Core Team (2012). R: A language and environment for statistical computing (R Foundation for Statistical Computing, Vienna, Austria. ISBN 3-900051-07-0, URL: http://www.R-project.org/). Statistically significant p-values obtained from logistic regression analysis were corrected for multiplicity using simultaneous inference. A p-value of ≤0.05 was taken as statistically significant.

## Results

The ID genotype frequency of the *ACE* polymorphism rs4646994 was significantly different between DR, DNR and healthy controls. Among the sub-clinical classes the ID frequency was higher in NPDR than PDR. The multivariate-gender adjusted logistic regression analysis resulted in significant association of ID genotype with the development of DR (OR 1.87 [95%CI = 1.043–3.36], p = 0.00883) and the sub-clinical class NPDR (OR 2.25 [95%CI = 1.098–4.62], p = 0.00584), which remained significant even after the correction of the data by simultaneous inference (DR p = 0.03057 and NPDR p = 0.02054, Table 2). However no association was observed for II and DD genotypes of *ACE* polymorphism with DR (Table 2).

**Table 2:** Logistic regression analysis for rs4646994 and rs1799768 in *ACE* and *PAI* genes in Pakistani type 2 diabetic cohort.

Genotype *ACE* rs11258194	DR Est.	Z value	OR (95%CI)	p value/ *p value	DNR Est.	Z value	OR (95%CI)	p value	PDR Est.	Z value	OR (95%CI)	p value	NPDR Est.	Z value	OR (95%CI)	p value/ *p value
**II**	--	--	--	--	--	--	--	--	--	--	--	--	--	--	--	--
**ID**	0.63	2.62	1.87 (1.04–3.36)^a^	**0.00883/** * **0.03057**	0.03	0.13452	1.03 (0.66–1.60)^a^	0.89	0.37	0.97	1.45 (0.78–2.70)^a^	0.24	0.81	2.76	2.25 (1.10–4.62)^a^	**0.00584/** * **0.02054**
**DD**	0.26	0.87	1.29 (0.62–2.70)^a^	0.79	0.30	1.14875	1.35 (0.81–2.26)^a^	0.25	0.27	0.69	1.31 (0.61–2.79)^a^	0.49	0.25	0.65	1.28 (0.50–3.25)^a^	0.91
**Allele Frequency**																
**I**	--	--	1.21(0.89–1.65)^b^	0.20	--	--	1.17(0.87–1.57^b^	0.27	--	--	1.19(0.81–1.76)^b^	0.35	--	--	1.24 (0.87–1.78)^b^	0.22
**D**	--	--			--	--			--	--			--	--			--	--	--	--	--	--	--	--
**Genotype *PAI-1* rs1799768**																
**5G/5G**	--	--	--	--	--	--	--	--	--	--	--	--	--	--	--	--
**4G/5G**	0.12	0.49	1.13(0.70–1.82)^a^	0.62	0.03	0.14370	1.03 (0.66–1.63) ^a^	0.89	0.25	0.77	1.28 (0.68–2.41)^a^	0.44	0.02	0.07	1.02 (0.57–1.83)^a^	0.94
**4G/4G**	-0.00007	-0.00025	0.99 (0.57–1.75)^a^	0.99	0.047	0.17520	1.05(0.62–1.77)	0.86	-0.23	-0.57	0.79 (0.36–1.74)^a^	0.56	0.14	0.41	1.15 (0.59–2.22)^a^	0.68
**Allele Frequency**																
**5G**	--	--	1.10(0.86–1.63)^b^	0.54	--	--	1.10(0.75–1.61)^b^	0.92	--	--	1.00(0.68–1.47)^b^	0.71	--	--	--	--
**4G**	--	--			--	--			--	--			--	--	--	--	--	--	--	--	--	--

^a^Gender and sub-clinical class (NPDR and PDR) adjusted odd ratio (OR) and 95% confidence interval (95%CI) from multivariate logistic regression analysis

^b^OR and (95%CI) from univariate logistic regression analysis

*p, corrected p value

The multivariate logistic regression analysis of the *PAI-1* polymorphism rs1799768 genotype data did not show association (p>0.05) of the risk allele 4G with the development of DR or any of its sub-clinical types in the studied cohort (Table 2).

## Discussion

In the current study of Pakistani T2DM subjects genetic association was studied of single nucleotide polymorphisms (SNPs) of *ACE* and *PAI-1*, which belong to the Renin-Angiotensin System (RAS) and Plasminogen Activator System (PAS), respectively, we observed significant association of *ACE* polymorphism with DR and NPDR. Worldwide different *ACE* and *PAI-1* SNP based case-control association studies have implicated ethnicity as an important disease susceptibility risk factor [12], not only for diabetes but also other diseases including myocardial infarction [11], schizophrenia [13], cardiovascular disease [14], dental caries [15], etc. ACE is a key regulator of RAS, which catalyzes the conversion of angiotensin I to angiotensin II. Several components of RAS such as ACE, pro-renin, renin, angiotensinogen, angiotensin I and II have been shown to be expressed in the human eye [16], but pathophysiological importance of RAS with respect to DR remains unclear. In the present study, a significant *ACE* genotype association was found with the onset of DR in Pakistan. These results are contradictory to some of the other studies in which no significant association of ID polymorphism was found with the development and progression of retinopathy in subjects with T1DM and T2DM [17, 18]. Nagi et al. [19] also did not observe any association of the *ACE* ID polymorphism in the Caucasian population when the genotypes of healthy non-diabetic controls were compared with TIDM and T2DM patients suffering from retinopathy including NPDR and PDR [19]. In addition, Gutierrez et al. [20] did not find any association of the polymorphism in a Mediterranean population suffering from T2DM.

However, in the current study because of the significantly higher frequency of heterozygous ID genotype in DR cases as compared to DNR and control, the ID genotype was found to be associated with the susceptibility of development of retinopathy in the Pakistani diabetics. As opposed to this Nikzamir et al. [21] found the homozygous DD risk genotype to be more common in DR subjects in the Iranians [21]. In the current study, *ACE* ID polymorphism was also found to be significantly associated with NPDR, while no significant association was observed in the case of PDR. This is in contrast to the previously reported significant association of ID polymorphism in Chinese [22] T2DM subjects with PDR. Results obtained in the Pakistani population therefore suggest a probable role of *ACE* in the development of the disease at early stages of retinopathy (NPDR), which is in contrast to the other studies, where significant association was found with advanced stages of retinopathy i.e. PDR. A reason for not finding an association of RAS with advanced stages of DR in the current study could be inadequate number of patients in the PDR group or ethnic variations between Pakistani and other populations, in addition there might be other pathophysiological mechanisms along with environmental factors that are also possibly involved in disease pathogenesis. Clinical and experimental studies on the pathophysiological role of RAS in DR underscores vasoconstriction, inflammation, oxidative stress, ischemia, angiogenesis, cell proliferation and fibrosis to be the major tissue responses of retinal microvasculature under hyperglycemic conditions that lead to retinal dysfunction in DR patients [23]. The RAS components angiotensin I and II, ACE, ACE2 and Angiotensin II type 1 receptor (AT1R) levels are reported to be higher in serum and eyes of diabetic rats [24]. Though the conversion of angiotensin I to angiotensin II by ACE is a critical step in glucose metabolism, blood pressure and homeostasis, but angiotensin II is the prime modulator in RAS due to its localization in retinal microvasculature pericytes and endothelial cells where it imparts mitogenic effect to retinal endothelial cells [25] and directly influences the expression of epithelium-derived growth factors including vascular endothelial growth factor (VEGF) [26]. VEGF is an angiogenic growth factor, which under hyperglycemia-mediated oxidative stress and hypoxic conditions enhances the production of neovascularization by VEGF up-regulation through Ang II and accounts for the proliferative form of DR [16]. Thus it also seems that in the Pakistani diabetics although *ACE* ID polymorphism is involved in the development of retinopathy but probably other factors such as angiogenic pathway e.g. VEGF etc. are involved in disease progression to the more severe form i.e. PDR. This would indicate that retinopathy is not a simple process but probably involves a pathway in which the patient first develops the less severe form of retinopathy NPDR and only develops the more severe proliferative form PDR when other factors like angiotensin II start playing their role in disease progression.

PAS consists of characteristic serine protease activator Tissue Plasminogen Activator (t-PA) and its inhibitor PAI-1, where t-PA is involved in the conversion of plasminogen to plasmin causing degradation of metalloproteinase matrix [27]. PAI-1 is the inhibitor of t-PA and is thought to promote anti-fibrinolysis resulting in the growth of extracellular matrix by blocking the plasminogen conversion to plasmin [27]. Studies have demonstrated that several polymorphisms including rs1799768 (4G/5G SNP), rs2227631, rs6465787 and rs2227674 within the *PAI-1* locus influence PAI-1 levels in the serum [28, 29]. In the current study no association of the 4G/5G polymorphism of *PAI-1* was found with the development and progression of retinopathy in subjects with T2DM. Although, the “4G” polymorphism has been previously shown to be associated with higher risk of DR in the Pima Indians [30], but in the current study no association was observed between the “4G” allele and genotypes of 4G/5G polymorphism with DR and its clinical subgroups NPDR and PDR in the Pakistani population. Our results are in agreement with the studies conducted on Mediterranean’s [31], Chinese [32], Euro-Brazilians [33], Tunisians [34], Whites [35] and Slovenians [36] suffering from T2DM, which strengthen the fact that this SNP has no contribution in retinopathy in diabetic subjects.

In conclusion, this is the second report of DR genetic association from Pakistan after our first report on PPARγ association with PDR [37], the screening of SNPs in *ACE* and *PAI-1* from RAS and PAS pathways in the present study indicated possible association of only *ACE* with DR and NPDR susceptibility but not with PDR.
